# Serum CTRP9 Reflects Coronary Collateralization in Nondiabetic Patients with Obstructive Coronary Artery Disease

**DOI:** 10.1155/2022/8537686

**Published:** 2022-03-10

**Authors:** Ang Gao, Jinxing Liu, Yan Liu, Chengping Hu, Yong Zhu, Yujie Zhou, Hongya Han, Yingxin Zhao

**Affiliations:** ^1^Department of Cardiology, Beijing Anzhen Hospital, Capital Medical University, Beijing 100029, China; ^2^Department of Cardiology, Fuwai Hospital, National Center for Cardiovascular Diseases, Chinese Academy of Medical Sciences and Peking Union Medical College, China

## Abstract

**Aim:**

To explore the association between the serum C1q/tumor necrosis factor-related protein 9 (CTRP9) and the formation of coronary collateral circulation in obstructive coronary artery disease (CAD).

**Methods:**

A total of 206 patients who underwent coronary angiography at Beijing Anzhen Hospital and had epicardial arteries with at least 95% stenotic lesion were enrolled. Blood samples were taken after an overnight fasting before the coronary angiography. Serum CTRP9 level was measured using commercial enzyme linked immunosorbent assay (ELISA) kit. The development of coronary collateralization was determined according to the Rentrop classification system. Rentrop score 0-1 was graded as impaired or less-developed coronary collateralization (*n* = 54) while the Rentrop score 2-3 was defined as well-developed collateralization (*n* = 152).

**Results:**

Serum CTRP9 level was significantly higher in well-developed collateralization and diabetes groups (*P* < 0.001). To further explore the association between the CTRP9 level and coronary collateralization, the enrolled participants were divided into 3 tertiles according to the serum CTRP9 level. The prevalence of impaired coronary collateralization decreased stepwise with the increasing CTRP9 tertiles (*P* for trend <0.001). Multivariate regression analysis showed that the serum CTRP9 is independently associated with well-developed collateralization, with an OR (95% CI) of 4.49 (1.75-11.55) and 8.98 (2.75-29.35) in the tertiles 2 and 3, respectively. The following subgroup and receiver-operating characteristic (ROC) analysis also indicated that the diagnostic value of serum CTRP9 level for detecting the formation of collateralization persisted only in nondiabetic participants. Lastly, adding the serum CTRP9 into the baseline model could increase the diagnostic value of established model consisting of relevant factor for the discrimination of well-developed collateralization only in the nondiabetic group (*P* = 0.046).

**Conclusions:**

Serum CTRP9 reflects well-developed coronary collateralization in nondiabetic patients with obstructive CAD, and CTRP9 level ≥ 1.217 indicated a greater chance to forming well-developed coronary collaterals.

## 1. Introduction

CAD remained to be the leading cause of death worldwide [1]. Despite the great advance in the management and treatment of these patients, be it the optimal medical therapy (OMT) or invasive revascularization, there are still a certain number of patients suffering from recurrent refractory angina pectoris due to the diffuse coronary artery lesion, absent conduits used for coronary artery bypass grafting (CABG), or coronary distal vessel with small diameter that is ineligible for vascular revascularization [2]. Those patients continued to experience restricted physical capacity and declining quality of life. There are some alternative strategies for those patients, such as coronary sinus reducer implantation [3], spinal cord stimulation [4], or cardiac shock wave therapy [5]; yet, the treatment efficacy is still limited. Coronary collateralizations are networks of arterial-to-arterial anastomotic connections that have the potential to provide blood supply against the ischemia injury caused by severely stenotic or even occluded coronary vessels [6]. Well-developed coronary collateralization could exert myocardial salvaging effect, limit the ischemia territory, improve the cardiac function, and ameliorate the prognosis of patients [7]. Hence, therapies targeting at stimulating the coronary collateral growth (CCG) could be a promising strategy for those patients. However, a large proportion of patients in real clinical setting tended to develop impaired or less-developed coronary collateralization that limits the collateral capacity to provide sufficient blood flow in the event of coronary artery occlusion [8, 9]. The underlying mechanisms are still poorly understood.

CTRP9, defined as the closest paralog of adiponectin (APN) in the C1q/tumor necrosis factor-related protein (CTRPs) family, is highly expressed in the heart and the adipose tissue [10]. Similar to the APN not only in structure, CTRP9 has also been demonstrated to share high similarity in metabolic function with that of APN [11]. Previous studies have shown the strong association between the serum CTRP9 and the coronary atherosclerosis [12] and further elucidated the great potential of CTRP9 in cardiovascular protection via regulating endothelial dysfunction [11], modulating metabolic pathways [13, 14] and inhibiting vascular inflammation [15], which were thought as the important mechanisms influencing the process of CCG [6]. However, the association between the serum CTRP9 and the development of coronary collateralization has been largely unknown. Considering the great importance of well-developed coronary collateralization in alleviating symptoms and improving the quality of life for CAD patients not suitable for vascular revascularization, it is pertinent to examine the relationship between serum CTRP9 and the formation of collateralization in CAD patients and determine whether serum CTRP9 could be a promising therapeutic target for patients with impaired collateralization in different metabolic status.

## 2. Methods

### 2.1. Study Populations

This study retrospectively enrolled CAD patients who underwent coronary angiography between the 1^st^ October 2020 and the 30^th^ April 2021 at Beijing Anzhen Hospital. All participants had severe epicardial artery stenotic lesions defined as at least 95% luminal diameter stenosis. Exclusion criteria were as follows: (1) epicardial artery stenotic lesion < 95%, (2) history of coronary artery bridge grafting, (3) severe hepatic and renal dysfunction, (4) New York Heart Association (NYHA) III-IV or left ventricular ejection fraction (LVEF) < 30%, (5) type I diabetes mellitus, (6) chronic inflammatory or infectious disease, and (7) some basic demographic data or laboratory biomarkers were not available. Finally, a total of 206 participants were recruited into this study. Written informed consents were obtained from all participants, and all the clinical investigations were approved by the Ethics Committee of Beijing Anzhen Hospital and conducted under the principles of the Declaration of Helsinki.

The diagnosis of type II diabetes mellitus (T2DM), hyperlipidemia, and hypertension were based on the recommendations from American Diabetes Association [16], Third Report of the National Cholesterol Education Program [17], and the guideline from European Society of Hypertension/European society of Cardiology (ESC) [18]. Metabolic syndrome (MetS) was defined according to the Diabetes Branch of the Chinese Medical Association, using body mass index (BMI) as a replacement of the waist circumference, that included 3 or more metabolic abnormalities can be diagnosed [19]. The type of CAD was diagnosed according to the ESC guideline for the diagnosis and management of chronic coronary syndrome [1].

### 2.2. Coronary Angiography and Rentrop Classification System

Coronary catheterization was routinely performed via the radial or femoral access using 6 or 7 Fr diagnostic catheters. Examination and evaluation of the angiograms were performed by two experienced interventional cardiologists who were blinded for the intention of this study. The complexity of CAD lesion was determined according to the SYNTAX scoring system [20]. Coronary chronic total occlusion (CTO) lesion was defined as a coronary lesion with thrombolysis in myocardial infarction flow grade 0 for at least 3 months [21]. The Rentrop classification system was used to evaluate the formation of the coronary collateralizations: 0, no visible filling of the coronary collaterals; 1, filling of the side branch but without the filling of the epicardial artery; 2, partial filling of the epicardial artery via the collateral branch; and 3, complete filling of the epicardial artery. In patients with one or more coronary collaterals, the highest grading of the collateral was selected for the analysis. Rentrop scores 2-3 were classified as well-developed coronary collateralization [22].

### 2.3. Demographic and Laboratory Measurements

Demographic data and medical history like age, BMI, current smoker, and alcohol use were all collected from the Beijing Anzhen Hospital medical information system. Blood samples were obtained at the day of patient admission after an overnight fasting. Routine laboratory technique was used to determine serum level of blood cell count, creatinine, blood urea, uric acid, high-sensitivity C-reactive protein (hs-CRP), and lipid and glucose profiles. The blood samples used to measure the level of CTRP9 were taken via venous puncture and placed into tubes coated with heparin as an anticoagulant. Centrifuge samples for 15 minutes at 3000 rpm at 5°C within 30 minutes, the layer of the supernatant was carefully transferred into the other tubes. These samples were stored at -80°C until the final measurements. Serum CTRP9 was assessed by commercially available ELISA kit (BLUE GENE, Shanghai, China) in accordance with the manufacturers' instructions. The assay procedure was as followed: (1) secure the desired numbers of coated wells in the holder then add 100 *μ*L of samples to the appropriate well in the antibody pre-coated microtiter plate. (2) Add 100 *μ*L of phosphate buffer saline (PBS) in the blank control well. Add 50 *μ*L of conjugate to each well. (3) Cover and incubate the plate for 1 hour at 37°C. (4) Wash plate 5 times with diluted wash solution (350 *μ*L/well/wash) using an auto water. (5) After washing, invert plate and blot by hitting the plate onto absorbent paper until no moisture appears. (6) Add 50 *μ*L substrate A and 50 *μ*L substrate B to each well including blank control well, subsequently. (7) Cover and incubate for 10 minutes at 37°C. (8) Add 50 *μ*L of stop solution to each well including blank control well. (9) Determine the optical density (O.D.) at 450 nm using a microplate reader immediately. Estimated glomerular filtration rate (eGFR) was estimated using the creatinine-based Modification of Diet in Renal Disease (DMRD) formula [23].

### 2.4. Statistical Analysis

To explore the association between serum CTRP9 and the formation of coronary collateralizations, these participants were divided into 3 groups according to the CTRP9 tertiles. Continuous variables were expressed as the mean ± standard deviation (SD) for normally distributed continuous variables. Some laboratory measurements like fasting blood glucose, triglyceride, and hs-CRP were presented as median with interquartile for the distribution of these biomarkers that were highly skewed. The difference between tertiles was compared using the one-way ANOVA or Kruskal-Wallis *H* test for normally or nonnormally distributed continuous variables. The difference of categorical variables between tertiles was compared using the chi-square test or Fisher's exact test. Univariate logistic regression analysis was preliminarily performed to screen the potential factors correlated with the formation of coronary collateralization. *P* value <0.15 was considered clinically relevant. After adjustment for various confounding factors, the ORs and 95% CI of serum CTRP9 to discriminate well-developed coronary collaterals were calculated using weighted multivariate logistic regression analysis. Further subgroup analysis was constructed to evaluate the correlation between serum CTRP9 and the formation of coronary collateralization in different glycometabolic status. Lastly, ROC analysis was performed to compare the diagnostic value of serum CTRP9 for the well-developed coronary collateralization in those patients with diabetes or not. All statistical analyses were performed using SPSS 23.0 (SPSS, Inc. Chicago, IL, USA) and MedCalc 19.1 (MedCalc software, Belgium). Two-tailed *P* value <0.05 was considered statistically significant.

## 3. Results

### 3.1. Baseline Characteristic

The baseline characteristics of enrolled patients were shown in Table 1. The mean age of these participants was 57.24 ± 9.37 years old, and most of them were male (84.5%). The prevalence of current drinker, smoker, unstable angina pectoris (UAP), hypertension, T2DM, hyperlipidemia, and MetS was 23.3%, 40.3%, 65.5%, 64.1%, 17.0%, 83.0%, and 52.9%, respectively. Notably, the prevalence of T2DM and MetS raised stepwise with the increasing of CTRP9 tertiles (*P* < 0.05). No significant differences were found between tertiles in BMI (*P* = 0.384), heart rate (HR) (*P* = 0.702), systolic blood pressure (SBP) (*P* = 0.760), and diastolic blood pressure (DBP) (*P* = 0.990). As with the angiographic findings, the mean SYNTAX score was 17.91 ± 7.87. Most of participants came with multivessel disease (79.6%). About a half of the diseased arteries related to the formation of coronary collateralization were located in the right coronary artery (RCA) (47.5%). There is a trend that the proportion of CTO lesions increased stepwise from the lowest CTRP9 tertile to the highest one (*P* = 0.069).

### 3.2. Association between the Serum CTRP9 and Coronary Collateralization

**Figure 1:**
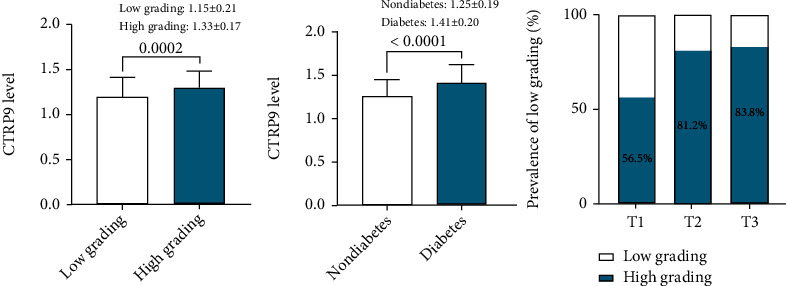
Comparison between the serum CTRP9 level in groups with different collateral grading; CTRP9_high grading_ vs. CTRP9_Low grading_: 1.33 ± 0.17 vs. 1.15 ± 0.21, with 95% CI of 0.05 to 0.17 (a) and glycometabolic status (b). The prevalence of low collateralization among patients stratified by serum CTRP9 tertiles (c). Abbreviation: CTRP9: C1q/tumor necrosis factor-related protein 9.

**Figure 2:**
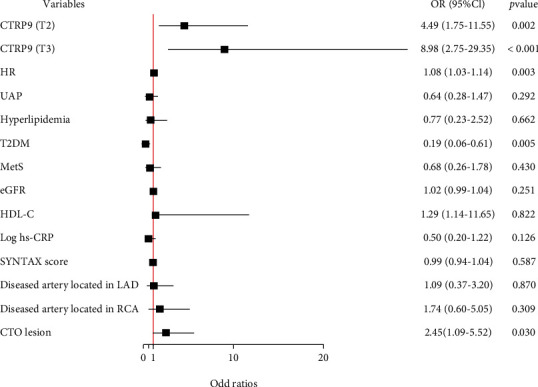
Forest plot of the results of multivariate logistic regression model exploring the association between serum CTRP9 and the formation of coronary collateralization in overall population. Abbreviations: CTRP9: C1q/tumor necrosis factor-related protein 9; HR: heart rate; UAP: unstable angina pectoris; T2DM: type II diabetes mellitus; MetS: metabolic syndrome; eGFR: estimated glomerular filtration rate; HDL-C: high-density lipoprotein cholesterol; Hs-CRP: high-sensitivity C-reactive protein; LAD: left anterior descending artery; RCA: right coronary artery; CTO: chronic total occlusion; OR: odd ratio; CI: confidence interval.

**Figure 3:**
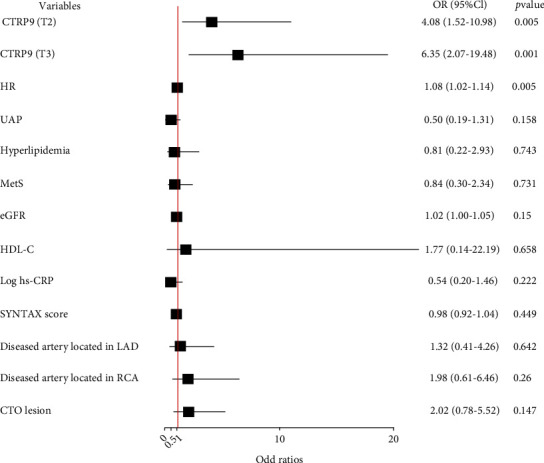
Forest plot of the multivariate logistic regression analysis model in nondiabetic patients with obstructive CAD exploring the association between serum CTRP9 level and the formation of collateralization. Abbreviations: CTRP9: C1q/tumor necrosis factor-related protein 9; HR: heart rate; UAP: unstable angina pectoris; MetS: metabolic syndrome; eGFR: estimated glomerular filtration rate; HDL-C: high-density lipoprotein cholesterol; hs-CRP: high sensitivity C-reactive protein; LAD: left anterior descending artery; RCA: right coronary artery; CTO: chronic total lesion; OR: odd ratio; CI: confidence interval.

**Figure 4:**
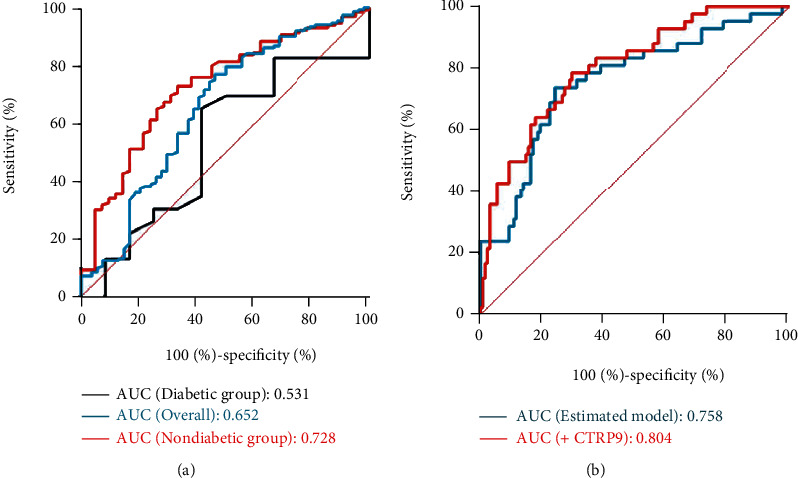
The diagnostic value of serum CTRP9 level in discriminating well-developed coronary collateralization in patients with different glycometabolic status (a). Different models for evaluating the formation of well-developed collateralization in nondiabetic patients (b). Established model include HR, type of CAD, hyperlipidemia, T2DM, MetS, eGFR, HDL-C, Log hs-CRP, diseased artery associated with the collateralization, SYNTAX score, and CTO lesion. Abbreviations: CTRP9: C1q/tumor necrosis factor-related protein 9; AUC: area under the curve; HR: heart rate; CAD: coronary artery disease; T2DM: type II diabetes mellitus; MetS: metabolic syndrome; eGFR: estimated glomerular filtration rate; HDL-C: high density lipoprotein cholesterol; hs-CRP: high-sensitivity C-reactive protein; CTO: chronic total occlusion.

The comparison of serum CTRP9 level between different groups and the prevalence of well-developed coronary collateralization stratified by the serum CTRP9 value were shown in Figure 1. The level of serum CTRP9 was significantly higher in patients with well-developed coronary collateralization (Figure 1(a)) and diabetes (Figure 1(b)) (*P* < 0.001). The prevalence of well-developed collateral increased from the lowest CTRP9 tertile to the highest one (tertile1 56.5% vs. tertile2 81.2% vs. tertile3 83.6%, *P* for trend <0.001) (Figure 1(c)). The results of the association between the serum CTRP9 and coronary collateralization after adjusting for various confounding factors were shown in Figure 2. The serum CTRP9 was modified as a categorical variable, the tertile 1 group was used as the reference, and the serum CTRP9 was independently associated with the well-developed coronary collateralization in the tertile 2 group (OR 4.49, 95% CI 1.75-11.55, *P* = 0.002) and tertile 3 group (OR 8.98, 95% CI 2.75-29.35, *P* < 0.001).  Figure 3 and Table 2 show that the diagnostic value of serum CTRP9 continued to persist in the nondiabetic group (tertile 2: OR 4.08, 95% CI 1.52-10.98, *P* = 0.005; tertile 3: OR 6.35, 95% CI 2.07-19.48, *P* = 0.001) but failed to substantiate that serum CTRP9 could independently discriminate the well-developed coronary collaterals in those with diabetes (tertile 2: OR 1.24, 95% CI 0.10-15.01, *P* = 0.865; tertile 3: OR 1.05, 95% CI 0.07-16.50, *P* = 0.975). The ROC curve in Figure 4(a) revealed the diagnostic values of serum CTRP9 to discriminate the formation of coronary collaterals in different glycometabolic status. A significant improvement in area under the curve (AUC) for serum CTRP9 to discern the well-developed collaterals can be found in the nondiabetic group (AUC in nondiabetic group 0.728 vs. AUC in overall participants 0.652, *P* < 0.05). Besides, Figure 4(b) shows that adding the serum CTRP9 to the established model consisting of HR, type of CAD, hyperlipidemia, MetS, diseased artery associated with the collateralization, CTO lesion, eGFR, high-density lipoprotein cholesterol (HDL-C), and Log hs-CRP can significantly the *C*-statistics for the discrimination of the coronary collateral growth (*P* = 0.046).

## 4. Discussion

The findings of this study are as follows: (1) the serum CTRP9 level is significantly higher in CAD patients with diabetes and well-developed coronary collateralization. (2) Serum CTRP9 is associated with well-developed coronary collaterals only in nondiabetic participants but not in those with diabetes. (3) Adding the serum CTRP9 into the baseline model consisting of various factors affecting the coronary collaterals could enhance the diagnostic value of discriminating well-developed collateralization in nondiabetic CAD patients.

The maturation of coronary collateralization into functional vessels has great clinical significance to preserving the normal cardiac function for its potency to provide an alternative source of blood supply to the ischemic myocardial territory supplied by the stenotic or even occluded vessels, especially present for at least 3 months [6]. Arteriogenesis, refers to the remodeling of pre-existing arterioles through wall thickening and diameter expanding into functional branches, is the main mechanism of collateral maturation in the presence of CAD [24]. The current study found a strong correlation between the serum CTRP9 level and well-developed coronary collateralization in the nondiabetic CAD patients, indicating that CTRP9 may involve in the process of arteriogenesis. The mechanism underlying could be explained by at least three aspects: mediating vascular inflammation, ameliorating endothelial dysfunction, and regulating oxidative stress [11, 15, 25–27]. In vivo studies have ensured the potential role of CTRP9 in inhibiting the expression of proinflammatory cytokines in the vascular endothelium [15, 25]. Jung et al. for the first time found that CTRP9 could decrease the expression of NF-*κ*B-mediated proinflammatory genes and attenuate the cytokine-induced vascular inflammation via the AMP-activated protein kinase (AMPK) way [15]. Studies about whether CTRP9 could promote endothelial function have also delivered positive results [11, 27]. Yamaguchi et al. [27] found that CTRP9-knockout mice are more likely to experience impaired blood flow recovery and decreased capillary density after the unilateral hindlimb ischemic surgery, and recombinant CTRP9 treatment could increase the capillary networks and migration of endothelial cells via the AMPK way, indicating the potential of CTRP9 in promoting vascular revascularization in the event of ischemic events. Zheng et al. [11] further elucidated CTRP9 showed the strongest function of mediating vascular relaxation among the CTRPs family via adiponectin receptor-1/AMPK/eNOS/nitric oxide signaling pathway. Excessive oxidative stress induced by abnormal lipid and glucose metabolism is another reason for less-developed collateral branch [13, 26]. CTRP9 could attenuate high glucose-induced oxidative stress by decreasing the expression of reactive oxygen species (ROS) through the activation of AMPK/Nrf2 signaling pathway [13]. Oxidized low-density lipoprotein (ox-LDL) accumulation in vascular endothelium plays a pivotal role in endothelial dysfunction and coronary atherosclerosis. CTRP9 can ameliorate ox LDL-induced endothelial injury by activating antioxidant enzyme via the peroxisome proliferator-activated receptor *γ* co-activator 1*α*/AMPK pathway [26]. Besides, recent study has also found cholesterol efflux capacity, characterized by well-functioning HDL is also a determinant of forming well-developed collateral branch [28]. Notably, this study failed to find the association between HDL and CCG, which is in accordance with our findings in current study (Figure 2). Lei et al. substantiated that CTRP9 could upregulated the expression of proteins important for cholesterol efflux and thus promoting the process of CCG [14].

In current study, we found that the serum CTRP9 level is significantly higher in patients with diabetes (Figure 1(b)). Previous studies have shown the important role of CTRP9 in glucose metabolism and mediating insulin resistance (IR) [25, 29–32]. In animal studies, CTRP9 was found to have the potential to inhibit vascular inflammation, protect the integrity of vascular endothelium, and exert cardioprotective effect via attenuating the endoplasmic reticulum stress in diabetic mice [25, 29]. Conversely, targeted deletion of CTRP9 in mice would lead to an increase in food take and body weight and also result in peripheral IR [32]. However, the relationship between serum CTRP9 and the presence of diabetes and IR in human have always been controversial [30, 31]. Jia et al. [31] found that serum CTRP9 was significantly higher in those diabetic and obese patients and positively correlated with metabolic indicators, which is also reflected in current study. The mechanism of the increased CTRP9 expression in diabetic patients could be attributable to the compensatory response to the IR or hyperglycemia or defensive response to the metabolic stress [33]. However, another study conducted by Hwang et al. [30] showed serum CTRP9 was inversely associated with fasting glucose and homeostasis model assessment for IR (HOMA-IR), indicating increased CTRP9 could reflect favorable glucose metabolic status. Besides, we also found that serum CTRP9 failed to be a determinant of well-developed coronary collateral branch in the diabetic group (Table 2). The angiogenic growth factor therapy failed to achieve the same results animal studies have delivered in human [34]. One likely explanation could be that CAD patients in clinical settings are generally older and are more likely to have multiple complications like T2DM and MetS compared with animal models, and previous studies have shown that patients with metabolic disorders tended to have complicated coronary lesions and form impaired collateralizations [8, 9]. Whether CTRP9 could be a promising therapeutic target for diffuse CAD patients not suitable for vascular revascularization especially for those with diabetes still needed to be further investigated.

Several limitations should be acknowledged: (1) the study design can only allow us to detect the association but incapable of exploring whether there exists a causal relationship between the serum CTRP9 and the development of coronary collateralization. (2) Although relevant factors influencing the development of coronary collateralization were incorporated into the multivariate logistic regression analysis, the current study did not include other possible factors like inflammatory cytokines and duration of stenotic or occluded coronary lesion. (3) While the formation of coronary collateralization could be more accurately assessed by coronary flow index [35], the Rentrop score system is relatively easy to use in clinical practice. (4) The findings of the association between CTRP9 and development of collateralization in diabetic group may be easily affected by the relatively small samples (*n* = 35). (5) While the Rentrop score is easy to use in current clinical practice, it is limited to the angiographic findings. A functional examination, such as cardiac magnetic resonance (CMR), to evaluate the perfusion and myocardial vitality could add additional value in understanding the cardioprotective role of CTRP9.

## 5. Conclusion

In conclusion, this study for the first time showed that serum CTRP9 is independently associated with the formation of well-developed coronary collateralization in nondiabetic CAD patients. Therapies targeting at serum CTRP9 may be a promising strategy for diffuse CAD patients without diabetes not suitable for vascular revascularization.

## Figures and Tables

**Table 1:** Basic clinical characteristics of enrolled patients classified by serum CTRP9 level.

CTRP9	Total (*n* = 206)	T1 (*n* = 69)	T2 (*n* = 69)	T3 (*n* = 68)	*P* values
		(T1 ≤ 1.23)	(1.23 < T2 < 1.36)	(T3 ≥ 1.36)	
Demographic data					
Age (years)	57.24 ± 9.37	55.87 ± 9.98	58.45 ± 9.24	57.41 ± 8.81	0.267
Male sex, *n* (%)	174 (84.5%)	58 (84.1%)	56 (81.2%)	60 (88.2%)	0.517
BMI (kg/m^2^)	26.55 ± 3.00	26.21 ± 2.95	26.49 ± 3.05	26.94 ± 3.00	0.384
SBP (mmHg)	137.64 ± 17.46	136.36 ± 16.72	138.25 ± 17.83	138.31 ± 18.00	0.760
DBP (mmHg)	80.78 ± 10.13	80.86 ± 9.43	80.64 ± 9.81	80.84 ± 11.23	0.990
HR (bpm)	72.59 ± 8.87	71.87 ± 7.94	72.84 ± 9.28	73.07 ± 9.40	0.702
Alcohol use, *n* (%)	48 (23.3%)	19 (27.5%)	14 (20.3%)	15 (22.1%)	0.577
Current smokers, *n* (%)	83 (40.3%)	27 (39.1%)	26 (37.7%)	30 (44.1%)	0.723
Types of CAD					
UAP, *n* (%)	135 (65.5%)	52 (75.4%)	42 (60.9%)	41 (60.3%)	0.108
SAP, *n* (%)	71 (34.5%)	17 (24.6%)	27 (39.1%)	27 (39.7%)	0.108
Medical history					
Hypertension, *n* (%)	132 (64.1%)	47 (68.1%)	44 (63.8%)	41 (60.3%)	0.633
T2DM, *n* (%)	35 (17.0%)	1 (1.4%)	10 (14.5%)	24 (35.3%)	<0.001^∗^
Hyperlipidemia, *n* (%)	171 (83.0%)	55 (79.7%)	53 (76.8%)	63 (92.6%)	0.032^∗^
MetS, *n* (%)	109 (52.9%)	30 (43.5%)	35 (50.7%)	44 (64.7%)	0.041^∗^
Laboratory measurement					
MONO	0.38 ± 0.13	0.40 ± 0.13	0.38 ± 0.13	0.36 ± 0.11	0.226
eGFR	95.54 ± 14.88	95.65 ± 14.29	92.84 ± 16.56	98.18 ± 13.31	0.110
CREA, mmol/L	74.09 ± 15.29	74.88 ± 14.90	75.43 ± 17.13	71.94 ± 13.60	0.359
UREA, mmol/L	5.03 ± 1.31	5.03 ± 1.30	5.22 ± 1.22	4.85 ± 1.40	0.251
UA, mmol/L	353.32 ± 91.67	355.06 ± 97.98	364.23 ± 95.65	340.49 ± 79.95	0.313
FBG, mmol/L	5.36 (4.92-6.22)	5.36 (5.01-6.09)	5.49 (4.95-6.32)	5.23 (4.77-6.35)	0.684
TC, mmol/L	4.25 ± 1.18	4.21 ± 0.99	4.32 ± 1.27	4.23 ± 1.29	0.839
TG, mmol/L	1.52 (1.13-2.13)	1.49 (1.07-2.19)	1.53 (1.07-2.03)	1.55 (1.18-2.21)	0.738
HDL-C, mmol/L	1.01 ± 0.22	1.04 ± 0.23	1.05 ± 0.25	0.96 ± 0.16	0.030^∗^
LDL-C, mmol/L	2.46 ± 1.01	2.38 ± 0.82	2.49 ± 1.08	2.51 ± 1.12	0.703
Non-HDL-C, mmol/L	3.23 ± 1.14	3.17 ± 0.97	3.27 ± 1.21	3.25 ± 1.25	0.822
Hs-CRP	1.18 (0.65-2.47)	1.29 (0.61-2.91)	1.13 (0.71-2.52)	1.12 (0.66-2.02)	0.811
Cardiovascular medications					
Antiplatelet agents, *n* (%)	175 (85.0%)	61 (88.4%)	59 (85.5%)	55 (80.9%)	0.425
ACEI/ARB, *n* (%)	52 (25.2%)	18 (26.1%)	13 (18.8%)	21 (30.9%)	0.284
CCB, *n* (%)	64 (31.1%)	26 (37.7%)	21 (30.4%)	17 (25.0%)	0.277
*β* receptor blocker, *n* (%)	98 (47.6%)	37 (53.6%)	32 (46.4%)	29 (42.6%)	0.433
Nitrates, *n* (%)	106 (51.5%)	43 (62.3%)	33 (47.8%)	30 (44.1%)	0.084
Statins, *n* (%)	148 (71.8%)	53 (76.8%)	50 (72.5%)	45 (66.2%)	0.364
Coronary angiography data					
SYNTAX score	17.91 ± 7.87	19.18 ± 8.07	16.42 ± 6.91	17.99 ± 8.45	0.119
Number of the diseased vessels					0.644
One-vessel disease, *n* (%)	42 (20.4%)	12 (17.4%)	13 (18.8%)	17 (25.0%)	0.503
Two-vessel disease, *n* (%)	62 (30.1%)	19 (27.5%)	24 (34.8%)	19 (27.9%)	0.581
Three-vessel disease, *n* (%)	102 (49.5%)	38 (55.1%)	32 (46.4%)	32 (47.1%)	0.525
CTO lesions, *n* (%)	104 (50.5%)	27 (39.1%)	39 (56.5%)	38 (55.9%)	0.069
Location of the diseased artery					0.353
RCA, *n* (%)	98 (47.6%)	32 (46.4%)	29 (42.0%)	37 (54.4%)	0.339
LCX, *n* (%)	35 (17.0%)	9 (13.0%)	13 (18.8%)	13 (19.1%)	0.563
LAD, *n* (%)	73 (35.4%)	28 (40.6%)	27 (39.1%)	18 (26.5%)	0.165

^∗^ indicated the differences between groups were statistically significant. Abbreviations: CTRP9: C1q/tumor necrosis factor-related protein 9; BMI: body mass index; SBP: systolic blood pressure; DBP: diastolic blood pressure; HR: heart rate; CAD: coronary artery disease; UAP: unstable angina pectoris; SAP: stable angina pectoris; T2DM: type II diabetes mellitus; MetS: metabolic syndrome; MONO: monocyte count; eGFR: estimated glomerular filtration rate; CREA: creatinine; UREA: urea; UA: uric acid; FBG: fasting blood glucose; TC: total cholesterol; TG: triglyceride; HDL-C: high-density lipoprotein cholesterol; LDL-C: low-density lipoprotein cholesterol; Hs-CRP: high sensitivity C-reactive protein; ACEI: angiotensin-converting enzyme inhibitor; ARB: angiotensin receptor blocker; CCB: calcium channel blocker; CTO: chronic total occlusion; RCA: right coronary artery; LCX: left circumflex artery; LAD: left anterior descending artery.

**Table 2:** Multivariate logistic regression analysis for the discrimination of impaired collateralization in the diabetic group.

Variables	Univariate		Multivariate	
	OR (95% CI)	*P* value	OR (95% CI)	*P* value
UAP	1.42 (0.31-6.47)	0.653	0.58 (0.05-7.06)	0.671
HR	1.08 (0.97-1.20)	0.155	1.18 (0.95-1.46)	0.134
Hyperlipidemia	0.61 (0.06-6.55)	0.680	0.21 (0.01-55.82)	0.206
MetS	0.33 (0.03-3.18)	0.336	0.90 (0.02-47.66)	0.895
eGFR	1.00 (0.94-1.06)	0.908	0.95 (0.86-1.06)	0.355
HDL-C	0.64 (0.02-26.97)	0.816	0.07 (0.01-136.32)	0.483
Log hs-CRP	0.55 (0.10-3.02)	0.488	0.31 (0.02-4.84)	0.404
SYNTAX score	1.01 (0.93-1.11)	0.758	1.04 (0.90-1.19)	0.623
Diseased artery in LAD	0.39 (0.09-1.77)	0.222	0.07 (0.01-2.30)	0.136
Diseased artery in RCA	1.56 (0.38-6.36)	0.538	0.14 (0.01-3.60)	0.237
CTRP9 (T2)	1.67 (0.29-9.45)	0.564	1.24 (0.10-15.01)	0.865
CTRP9 (T3)	1.09 (0.21-5.76)	0.916	1.05 (0.07-16.50)	0.975

Abbreviations: UAP: unstable angina pectoris; HR: heart rate; MetS: metabolic syndrome; eGFR: estimated glomerular filtration rate; HDL-C: high-density lipoprotein cholesterol; Hs-CRP: high-sensitivity C-reactive protein; LAD: left anterior descending artery; RCA: right coronary artery; CTRP9: C1q/tumor necrosis factor-related protein 9.

## Data Availability

The dataset and materials mentioned above are available from the authors.

## Abbreviations

CTRP9: .C1q/tumor necrosis factor-related protein 9
CAD: Coronary artery disease
ELISA: Enzyme linked immunosorbent assay
ROC: Receiver-operating characteristics
AUC: Area under the curve
OR: Odds ratio
CI: Confidence interval
SD: Standard deviation
OMT: Optimal drug therapy
CABG: Coronary artery bridge grafting
CCG: Coronary collateral growth
APN: Adiponectin
NYHA: New York Heart Association
LVEF: Left ventricular ejection fraction
T2DM: Type II diabetes mellitus
eGFR: Estimated glomerular filtration rate
DMRD: Modification of Diet in Renal Disease
ESC: European Society of Cardiology
MetS: Metabolic syndrome
BMI: Body mass index
CTO: Chronic total occlusion
Hs-CRP: High-sensitivity C-reactive protein
UAP: Unstable angina pectoris
HR: Heart rate
SBP: Systolic blood pressure
DBP: Diastolic blood pressure
RCA: Right coronary artery
IR: Insulin resistance
HOMA-IR: Homeostasis model assessment for IR
HDL-C: High-density lipoprotein cholesterol
ox-LDL: Oxidized low-density lipoprotein
ROS: Reactive oxygen species
AMPK: AMP-activated protein kinase
CMR: Cardiac magnetic resonance
PBS: Phosphate buffer saline.
